# A VLP Library of C-Terminally Truncated Hepatitis B Core Proteins: Correlation of RNA Encapsidation with a Th1/Th2 Switch in the Immune Responses of Mice

**DOI:** 10.1371/journal.pone.0075938

**Published:** 2013-09-23

**Authors:** Irina Sominskaya, Dace Skrastina, Ivars Petrovskis, Andris Dishlers, Ieva Berza, Maria Mihailova, Juris Jansons, Inara Akopjana, Irina Stahovska, Dzidra Dreilina, Velta Ose, Paul Pumpens

**Affiliations:** Protein Engineering Department, Latvian Biomedical Research and Study Centre, Riga, Latvia; Institut National de la Santé et de la Recherche Médicale, France

## Abstract

An efficient pBR327- and Ptrp-based *E. coli* expression system was used to generate a large-scale library of virus like particles (VLP) formed by recombinant hepatitis B virus (HBV) core (HBc) protein derivatives. To construct the library, the gene of HBc protein of the genotype D/subtype ayw2 virus was gradually truncated from the 3`-end and twenty-two HBc variants (with truncation up to 139 aa) were expressed at high levels. The proteins were purified by salt precipitation and gel filtration. Background RNA binding was observed for VLPs formed by HBc1-149, which lacked all C-terminal Arg blocks, and the addition of three Arg residues (HBc1-152) only slightly increased RNA binding. The presence of two Arg blocks (proteins HBc1-162 and HBc1-163) resulted in approximately half of the typical level of RNA binding, and the presence of three blocks (protein HBc1-171) led to approximately 85% of the typical level of binding. Only a small increase in the level of RNA binding was found for the HBc1-175 VLPs, which contained all four Arg blocks but lacked the last 8 aa of the full-length HBc protein. VLPs containing high levels of RNA had higher antigenicity according to an ELISA with anti-HBc mAbs than the VLPs formed by HBc variants without C-terminal Arg blocks and lacking RNA. The results indicate that the VLPs were stabilised by nucleic acids. The immunogenicity in BALB/c mice was comparable for VLPs formed by different HBc proteins, but a clear switch from a Th1 response to a Th2 response occurred after the loss of encapsidated RNA. We did not observe significant differences in lymphocyte proliferation *in vitro* for the tested VLP variants; however, the loss of RNA encapsidation correlated with a decreased level of IFN-γ induction, which is a measure of the potential CTL activity of immunogens.

## Introduction

Virus-like particles (VLPs) have been used extensively to present epitopes and other functional oligopeptides in the design of potential vaccines and gene delivery tools to target cells (for a review, see 1,2). VLPs have also been investigated as prospective nanocontainers for low-molecular-weight drugs and inorganic nanoparticles [1,3].

VLPs formed by the hepatitis B virus (HBV) core (HBc) protein have attracted interest worldwide, both as an object for structural investigations and as a highly efficient carrier of foreign insertions [4]. The HBV gene C, which encodes the HBc protein, has been expressed in *E. coli* [5–8], the yeast *S. cerevisiae* [9,10] and *Pichia* *pastoris* [11,12], plants [13,14], and insect cells [15–17]. Variable amounts of HBc VLPs have been obtained using these systems, with *E. coli* remaining the dominant system for the expression of full-length or truncated HBc variants, commonly of 144 or 149 amino acids (aa) long which lack the nucleic acid binding domains. Typically, high-copy-number plasmids are used for HBc gene expression, and λP_L_, Ptac, PlacUV5, and P_T7_ are the most frequently used promoters with chemical or thermal induction.

The HBc protein, which is 183 aa long (except in HBV genotype A, in which it is 185 aa long), serves only as a structural molecule in the HBV nucleocapsid and possesses the ability to form dimeric units [7,18], which are further self-assembled into two forms of icosahedral particles: T=4 and T=3 particles, with diameters of 35 and 32 nm, respectively [19,20]. The three-dimensional structure of T=4 particles has been solved by X-ray crystallography [21], and a quasi-atomic model of the native T=3 shell has been calculated [22]. Although structural investigations have been performed using recombinant HBc particles produced mostly in *E. coli* cells, the structural data are valid for natural HBc particles because HBc particles from the livers of infected patients do not differ from recombinant particles with respect to both the presence and the ratio of the T=4 and T=3 forms [22]. In HBV virions, HBc exists only in the T=4 form [22,23].

The HBc protein consists of two mutually independent domains: the self-assembly (SA) domain (aa 1-140) and the protamine-like polyarginine (PA) domain (aa 150-183) [24]. The PA domain is responsible for the functions of HBc associated with viral replication: the encapsidation of the pregenomic RNA, the packaging of partially double-stranded genomic DNA [25], phosphorylation [26], and nuclear targeting [27], although the last function has been called into question recently [28]. The nucleic acid binding sites are located within four arginine blocks within the PA domain [29], and previous investigations have shown that the elimination of the PA domain results in a strong decrease in the intrinsic ability of HBc to encapsidate nonspecific RNA, mostly mRNAs, during heterologous expression in *E. coli* [30,31] and insect cells [32]. The loss of encapsidation activity is accompanied by two major discrepancies between C-terminally truncated HBc particles and the full-length HBc protein when these proteins are produced in *E. coli*. First, there is a substantial change in the T=4/T=3 ratio: the T=4 form is the predominant form for full-length HBc1-183, whereas the C-terminally truncated HBc1-140 protein forms mostly T=3 particles [32–34]. Second, the type of humoral response changes from a predominantly Th1 response, with the production of IgG2a antibodies, to a predominantly Th2 response, with the production of IgG1 antibodies [35,36].

The PA domain is conserved among HBV genotypes and has a rather flexible structure without any distinct tertiary structure, although no 3D data are available yet. The PA domain may be present inside [37] or outside the HBc particles [38,39]. The PA domain is separated from the SA domain by a hinge peptide, 141-STLPETTVV-149, which forms a mobile array and is involved in capsid morphogenesis and the management of the encapsidated nucleic acid [40,41].

In this study, an *E. coli*-based expression system envoving the high-copy-number plasmid pBR327 and Ptrp promoter was used to generate a VLP library in which individual VLPs were formed by HBc proteins differing in the length of the nucleic acid binding domain. This expression system allowed high levels of VLP synthesis without the use of chemical inducers. The effects of the C-terminal truncation of the HBc PA domain and the interdomain hinge on self-assembly, capsid stability, RNA and DNA binding, and, in particular, the ability of the purified HBc derivatives to induce specific B- and T-cell responses in Balb/C mice were studied using structurally different VLPs. Twenty-two truncated variants of the HBc protein were expressed, and VLP formation was observed for 21 of the variants. Our data confirmed the previous finding that HBc can tolerate C-terminal truncation up to aa 140 while maintaining assembly competence and clearly showed that at least two Arg blocks are necessary for substantial nucleic acid binding. The increases in RNA and DNA binding correlated with the increase in the length of the HBc protein. The change in the encapsidation ability of the truncated HBc derivatives correlated with the switch in the immune responses of Balb/C mice from a Th1 response, with the production of IgG2a antibodies, to a Th2 response, with the production of IgG1 antibodies.

## Materials and Methods

### Bacterial strains

*Escherichia coli* strain RR1 [F^-^ r_B_^-^ m_B_^-^ *leuB6 proA2 thi-1 araC14 lacY1 galK2 xyl-5 mtl-1 rpsL20* (Str^r^) *glnV44* Δ (*mcrC-mrr*)] was used for the cloning and selection of recombinant plasmids. For the expression of HBc genes, *E. coli* strains K802 (F^-^ r_K_^-^ m_K_^+^ *e14 McrA metB1 lacY1* [*or lacI-Y6*] *galK2 galT22 glnV44 mcrB*) and BL21 [F^-^ *ompT hsdS*_B_ (r_B_^-^ m_B_^-^) *gal dcm lon*] were used.

### Construction of plasmids for the expression of HBc gene variants

**Figure 1:**
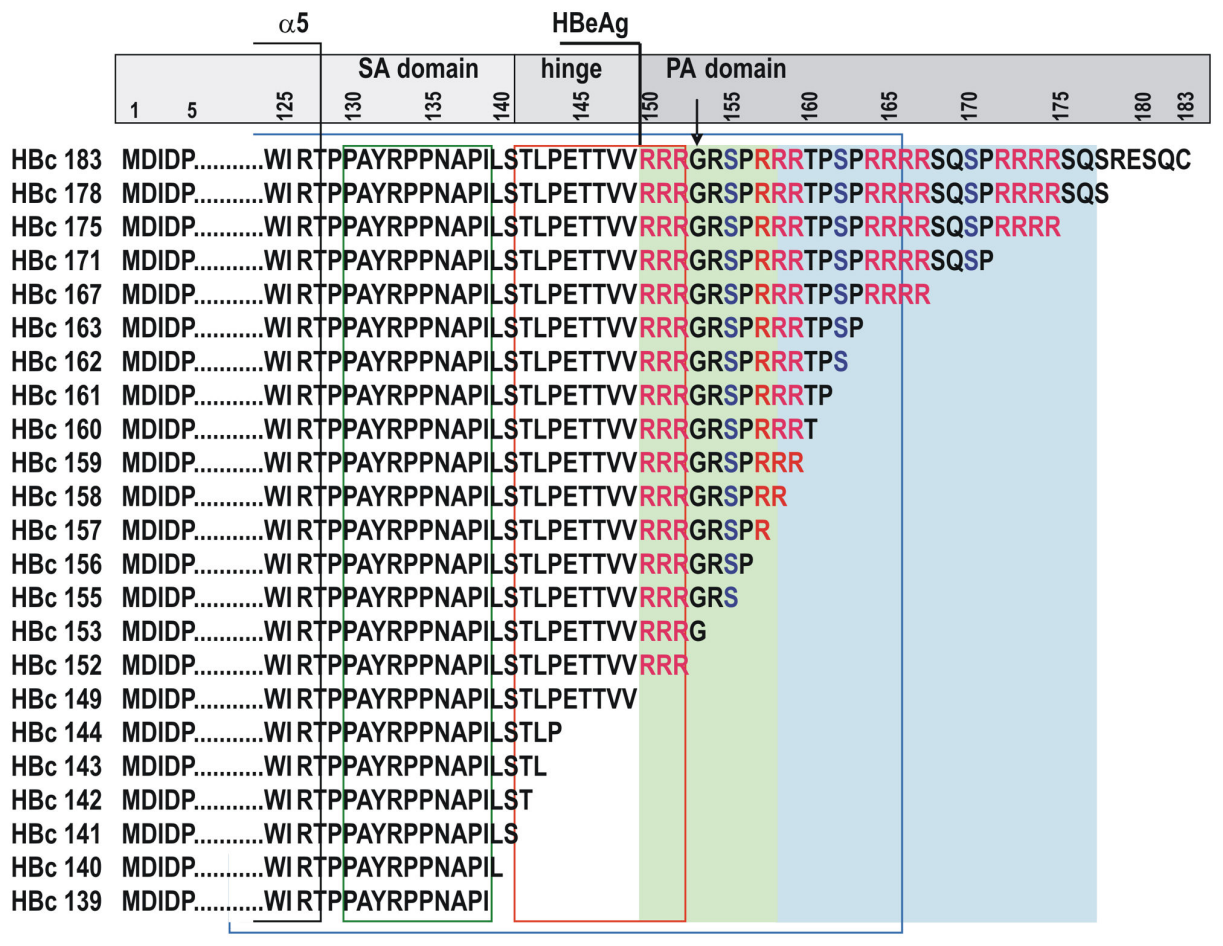
Schematic representation of the HBc-derived proteins constructed in the current study.

The HBc protein variants constructed in this study are shown in Figure 1. HBc gene deletion variants for the expression of HBc proteins ending at aa positions 139, 140, 141, 142, 143, 144, 149, 152, 153, 155, 156, 157, 158, 159, 160, 161, 162, 163, 167, 171, 175, and 178, 22 variants in total (Figure 1) were obtained using the plasmid pHBcI719 to generate the appropriate PCR fragments. The expression plasmid pHBcI719 was based on the pBR327 vector and ensured the expression of the full-length HBc gene under the control of Ptrp [42]. The HBc gene originated from the plasmid pHB320 containing the cloned genome of HBV (genotype D, subtype ayw2) [43]. The neomycin/kanamycin resistance gene was from the plasmid REP4 (Qiagen), The *E. coli* Ptrp promoter was from the HBc gene expression plasmid pGC74, as described in [5]. The upstream primer for cloning of HBc gene variants included an XbaI site, and the downstream primers included the stop codons TAGTAA at selected positions in addition to a BamHI site (the structure of primers is not shown). The amplified fragments were eluted from agarose gels using a gel extraction kit (Fermentas, Lithuania) and ligated with the large *XbaI/BamHI* fragment of the plasmid pHBcI719. The ligation products were used to transform *E. coli* RR1 cells. Plasmids were screened for the presence of the appropriate restriction sites (Fermentas, Lithuania), and the plasmid structures were confirmed by sequencing.

### Expression and purification of HBc proteins

For the expression of the HBc genes, two *E. coli* strains, K802 and BL21, were used. The K802 strain was used for the expression of longer HBc gene variants, including the full-length HBc gene, and the BL21 strain was used for the expression of the short and medium HBc gene variants. Protein expression was characterised by SDS-PAGE and subsequent Western blotting with the anti-HBc mAb 13C9 [44].

Cells were cultivated in Trp-deficient M9 salt medium supplemented with 1% casamino acids, 0.2% glucose (BD, USA), Ap, and Km (at 50 and 10 µg/mL, respectively) without additional induction of Ptrp. The aeration of the medium was ensured by incubating 750 mL Erlenmeyer flasks filled with 300 mL of medium on an orbital shaker at 200 rpm. The cultures were incubated for 14-16 h at 37°C, reaching a final OD_540_ of 5–8. The OD (optical density) correlated with the length of the expressed HBc gene: the lowest OD was observed for the full-length HBc gene. To assess HBc protein synthesis, 2 OD units of cells were pelleted and lysed in 200 µL of Laemmli buffer for 10 min at 100°C, and 10 µL of the lysate was subjected to SDS-PAGE. For the purification of the VLPs, 2 g of wet fresh cells were incubated for 30 min on ice in 8 mL of lysis buffer containing 50 mM Tris-HCl, pH 8.0, 5 mM EDTA, 50 µg/mL PMSF, 0.5 M urea, and 0.1% Triton X-100. The suspension was ultrasonicated five times at 22 kHz at 15 s intervals while keeping the cells on ice. After centrifugation at 10000 rpm for 30 min, ammonium sulphate was added to the supernatant at 33% saturation, and the mixture was incubated at 4°C overnight. The pellet obtained after centrifugation at 10000 rpm for 30 min was resuspended in 4 mL of PBS buffer containing 0.1% Triton X-100, 50 µM PMSF, and 0.5 M urea and loaded onto a Sepharose CL-4B column (2.5 x 85 cm). PBS buffer without Triton X100 was used for elution. HBc-containing fractions (detected by SDS-PAGE) were pooled, and the proteins precipitated by adding ammonium sulphate at 50% saturation for 20 h at 4°C. The pellet obtained after centrifugation at 10000 rpm for 30 min was resuspended in a minimal volume of PBS containing 0.5-1.0 M urea (the concentration of urea depended on the specific HBc derivative), and the suspension was dialysed overnight against 2000 volumes of PBS. Proteins with final concentrations of 2-10 mg/mL in 50% glycerol were stored at -20°C.

### Detection of protein and nucleic acids

The protein concentration was determined using the Bradford method. The specific RNA and DNA contents (μg/mg of protein) of the VLP preparations were estimated by using a Qubit fluorometer (Invitrogen, USA). The VLP preparations were compared with respect to the presence of protein and nucleic acids using native agarose gel electrophoresis (NAGE) and double radial immunodiffusion (DRI) according to the method of Ouchterlony [45] using rabbit polyclonal anti-HBc antibodies. For NAGE and DRI, 1% UltraPure agarose (Invitrogen, USA) in TBE buffer and 0.8% agarose M (Pharmacia Biotech, Denmark) in PBS buffer were used, respectively, with subsequent staining of the gels. The gels were first stained with EtBr (5 µL of a 10 mg/mL stock in 100 mL of PBS) and then with Coomassie Blue R-250 (60 µg/mL of Coomassie Blue R-250 in 10% acetic acid). For the Western blots, the mAb 13C9 [44] was used at a 1:1000 dilution. All chemicals were from Sigma Aldrich.

### EM analysis of core particles

Suspension of VLPs in PBS were adsorbed onto carbon-Formvar-coated copper grids and stained with 1% uranyl acetate. The grids were examined with a JEM-100C (JEOL Ltd., Tokyo, Japan) electron microscope at 80 kV.

### Antigenicity test

For the direct ELISA, 96-well microplates (Nunc, USA) were coated with the appropriate HBc proteins using 100 µL of protein solution (10 µg/mL in 50 mM sodium carbonate buffer, pH 9.6) per well. The plates were incubated with the protein solution overnight at 4°C. After the plates were blocked with 1% BSA in PBS for 1 h at 37°C, serial dilutions of mAbs were added to the wells, and the plates were incubated at 37°C for an additional 1 h. After washing three times with PBS containing 0.05% Tween-20, 100 µL of horseradish peroxidase conjugated anti-mouse antibody (Sigma, USA) was added at a 1:10,000 dilution. After incubation at 37°C for 1 h, the plates were washed, and OPD substrate (Sigma, USA) was added for colour development. A Multiscan (Sweden) was used to measure the absorbance at 492 nm. The VLP antigenicities determined using the mAbs C1-5 [46] and 10E11 [44] were compared using this ELISA. The end-point titres were defined as the highest mAb dilution that resulted in an absorbance value three times greater than that of the nonspecific control.

For the competitive ELISA, 96-well microplates were coated with full-length HBc using 100 µL of protein solution (10 µg/mL) per well. After the plates had been coated, 50 µL aliquots of serial dilutions of competing proteins and 50 µL of the anti-HBc mAb C1-5 were added to the wells simultaneously. The dilution of the mAb with an OD_492_ value within the range of 0.5-1.0 in the control sample without competing protein was used. After incubation at 37°C for 1 h, the microplates were processed as described above. The per cent inhibition (I%) of antibody binding by the competing protein was calculated as follows:

I %= [(OD_492_ test sample – OD_492_ negative control) / (OD_492_ positive control - OD_492_ negative control)] x 100.

The molar amounts of the proteins necessary for 50% inhibition (I_50_) were calculated.

### Immunogenicity of the HBc VLPs

Female Balb/C mice, 6-8 weeks of age, obtained from Latvian Experimental Animal Laboratory, Riga Stradins University, were maintained at the Biomedical Research and Study Centre under pathogen-free conditions. The experiments were approved by the Latvian Animal Protection Ethics Committee and the Latvian Food and Veterinary service, permission No. 31/23.10.2010. Groups of five mice were immunised subcutaneously with 25 µg of protein (diluted in 0.2 mL of PBS) per mouse on days 0, 14, and 28. To assess the humoral response, the animals were bled on days 28 and 42, and the anti-HBc titres in the sera were determined with a direct ELISA using plates coated with the full-length HBc protein. For the T-cell proliferation tests, the mice were sacrificed, and their spleens were obtained on day 42. The IgG1 and IgG2a subsets in the sera of the immunised mice were detected with isotype specific ELISAs using a mouse monoclonal antibody isotyping reagent (ISO-2, Sigma, USA) and an anti-goat/sheep IgG peroxidase conjugate (Sigma, USA). The end-point titres were defined as the highest serum dilution that resulted in an absorbance value three times greater than that of the control sera obtained from unimmunised mice.

### T-cell proliferation assay and cytokine test

Murine splenocytes were harvested using red blood cell lysis buffer (Sigma, USA). Lymphocyte suspensions were prepared (5 x 10^6^ cells/mL) and co-cultured in RPMI 1640 (Gibco, Germany) with full-length HBc (1.0 µg/mL). The plates were incubated for 96 h, and 1 µCi [^3^H]-thymidine (Amersham, UK) was added for the last 18 h. The level of [^3^H]-thymidine incorporation was measured using a liquid scintillation β-counter. The proliferative response is expressed as the stimulation index (SI) calculated as the mean cpm of HBc-stimulated cells divided by the mean cpm of unstimulated cells.

For the detection of the cytokines IL-2 and IFN-γ in HBc-stimulated cell cultures, the supernatants of the cell cultures were removed at 24 h and 48 h after starting the T-cell proliferation test for IL-2 and IFN-γ, respectively. The analysis of the presence of cytokines in the cell supernatants was performed using the Mouse IFN-γ ELISA Set and the Mouse IL-2 ELISA Set (OptEIA, BD, USA) according to the manufacturer’s instructions.

## Results and Discussion

In this study, we generated the most representative library of HBc proteins (Figure 1) by gradually truncating both the hinge and PA domains, which resulted in different numbers of nucleic acid binding sites at the C-terminus of HBc. The proteins were purified to compare their VLP-forming ability, stability, and recovery after standard purification procedures, such as precipitation by ammonium sulphate and gel filtration chromatography. The corresponding VLPs were investigated to determine the amount of incorporated nonspecific host (*E. coli*) nucleic acids, antigenicity, and B- and T-cell immunogenicities in mice of the VLPs were compared.

The expression levels of HBc proteins varied in the range of 5-10% of the total cellular protein depending on the HBc variant, with a tendency for higher expression for shorter HBc variants, although a high level of expression was achieved for all HBc variants.

**Figure 2:**
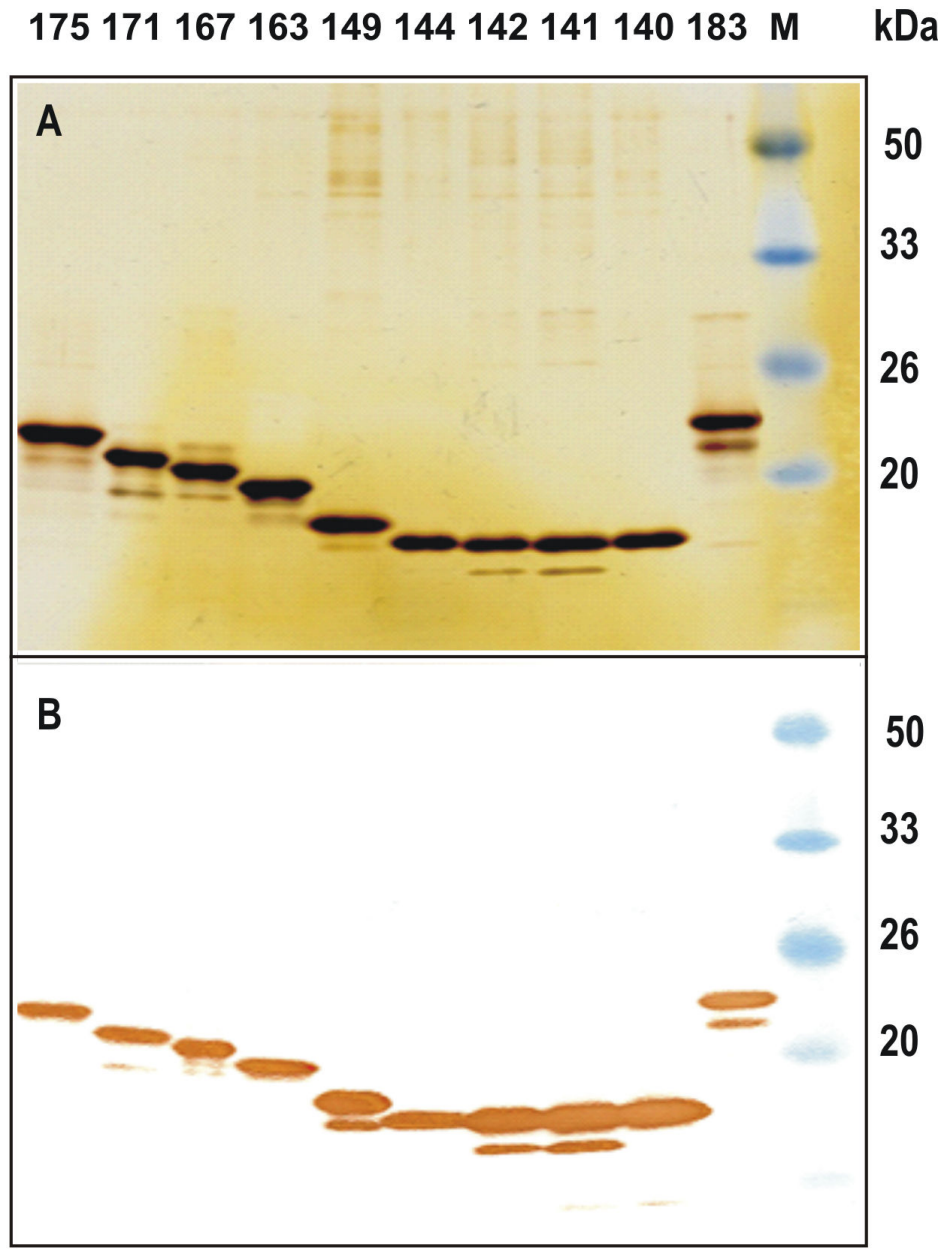
SDS-PAGE analysis of purified HBc proteins. **2A** - Silver-stained gel; **2B** - Western blot. Normalised amounts of protein (10 µg) were loaded onto the gel. HBc proteins: 1 -1-175, 2 -1-171, 3 -1-167, 4 -1-163, 5 -1-149, 6 -1-144, 7 -1-142, 8 -1-141, 9 -1-140, and 10 -1-183. For the Western blot, the anti-HBc mAb 13C9, which recognises the epitope 134-PPNAPIL-140 [44], was used. The Prestained Protein Molecular Weight Marker (Fermentas, Lithuania) was used (M).

**Figure 3:**
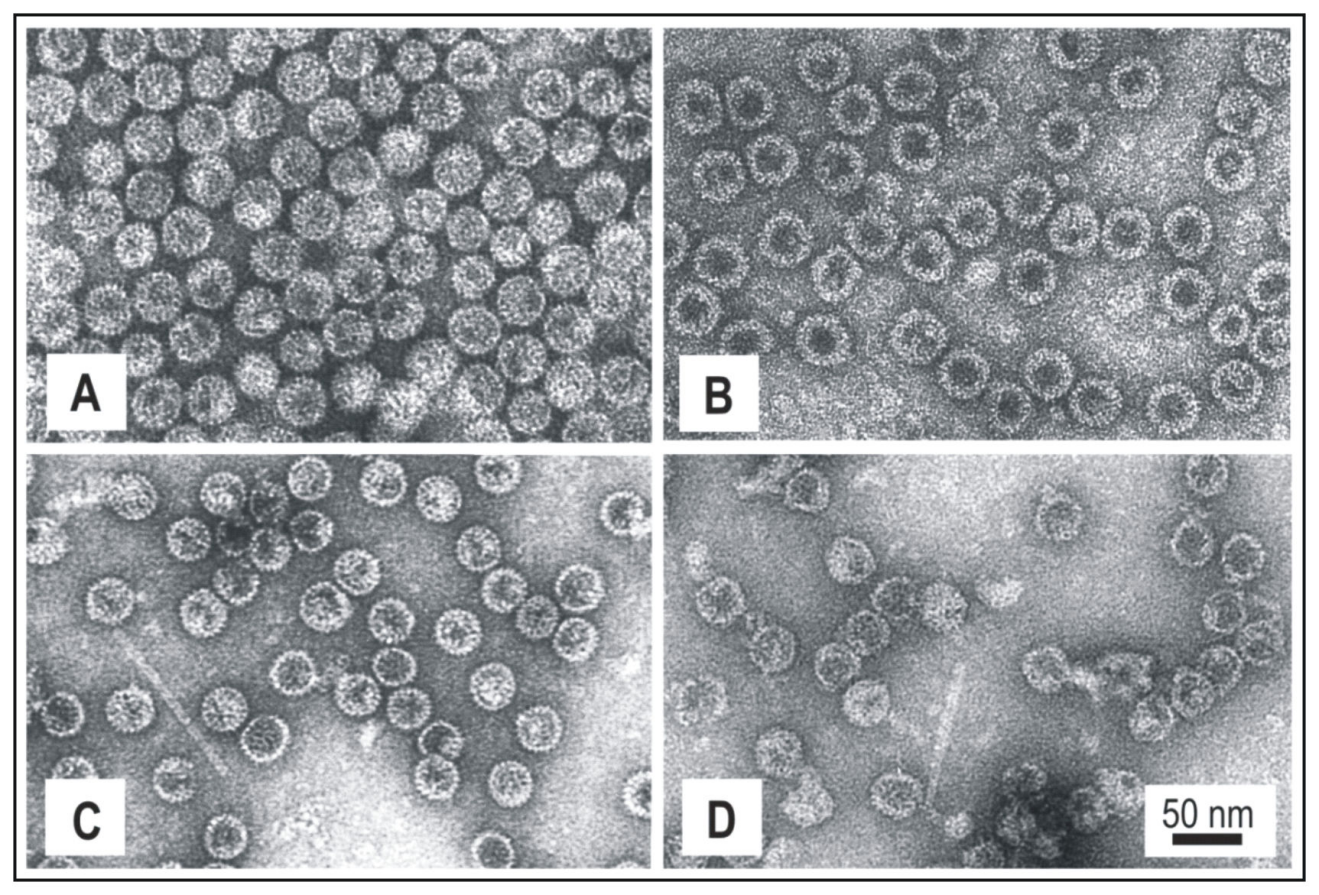
Electron microscopy analysis of purified HBc-VLPs. A – HBc1-183, B – HBc1-161, C – HBc1-152, and D – HBc1-141. Scale bar, 50 nm.

For the purification of HBc proteins in the form of VLPs, fractionation with ammonium sulphate was used, followed by gel filtration. Approximately 10-20 mg of purified VLPs per gram of wet cells was the average yield of VLPs in all cases. The purity level of VLP preparations was estimated to be in the range of 85-95%. The VLPs formed by short HBc variants appeared to be more contaminated with host proteins, possibly due to the incorporation of host proteins within the capsids. A representative part of the normalised HBc proteins analysed by SDS-PAGE with silver-staining and Western blotting is shown in Figure 2. Notably, the HBc protein variants lacking Arg blocks (HBc1-140, 1-141, 1-142, 1-149) were better transferred to the blotting membrane. EM analysis confirmed the higher stability and quality of the VLPs formed by non-truncated HBc (Figure 3).

As previously found, the HBc1-139 variant did not form particles [24], although its expression level and solubility were comparable to those of other HBc variants. A “mini-C” band was visible for most of the HBc variants in the silver-stained gel and the immunoblot prepared with mAb 13C9, which recognises the epitope 134-140 (Figure 2). The distance of the mini-C band from the basic HBc band in all cases in which it was present was similar, indicating the possibility that the HBc proteins were specifically cleaved near the N-terminus; however, the lack of this band in some cases (HBc1-144, HBc1-140) indicates that there were conformational differences among the VLPs formed by different HBc proteins. The non-particulate HBc1-139 variant showed the same proteolytic stability as other proteins with the presence of the same mini-C band.

**Figure 4:**
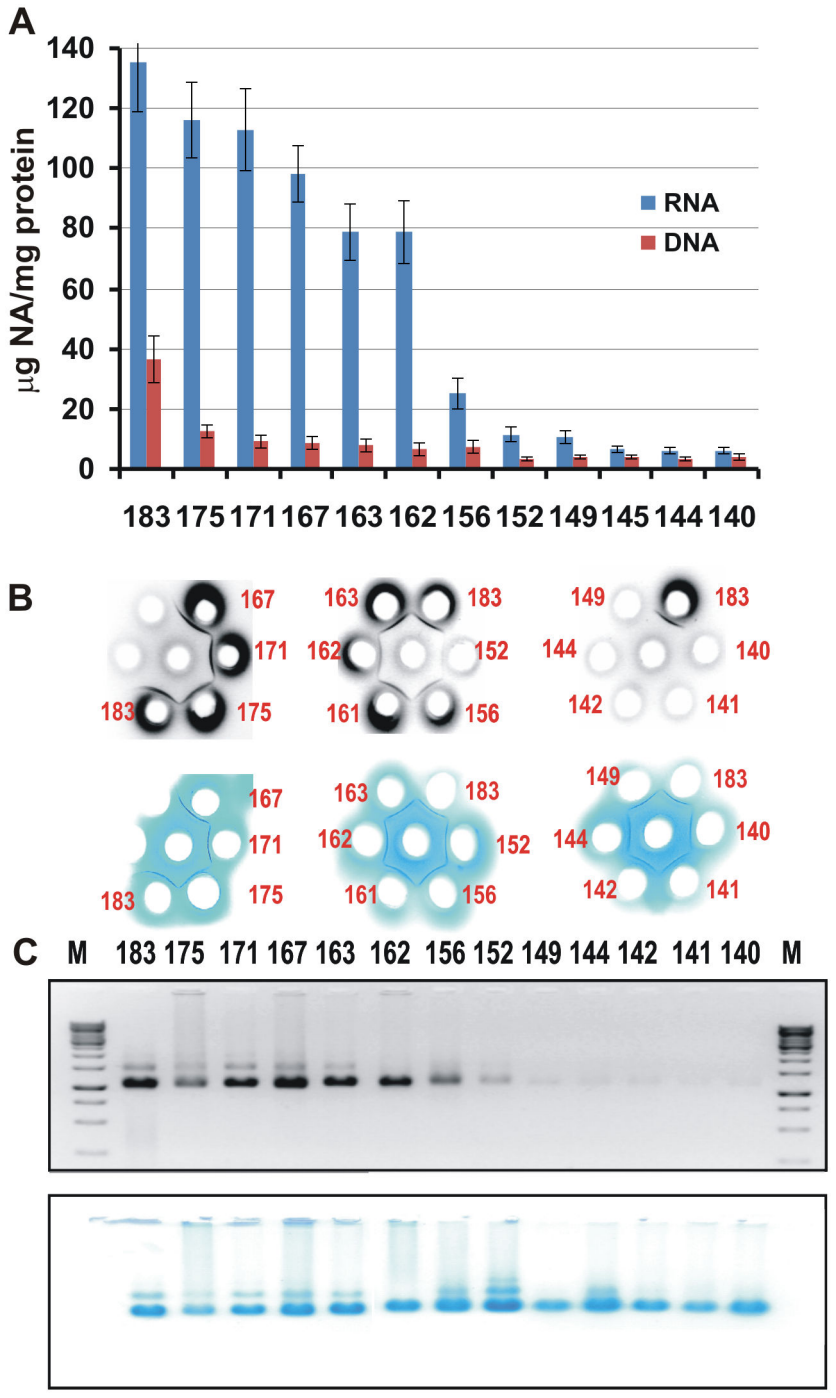
Nucleic acid binding abilities of the VLPs formed by HBc proteins of different lengths. **4A** - The RNA/protein and DNA/protein ratios (µg/mg) measured using a Qubit fluorometer. The results represent the mean values (± SD) of three separate experiments; **4B** - Double radial immunodiffusion using Ouchterlony method (0.8% agarose gel in PBS); the gels were stained with EtBr (upper panel) and Coomassie blue R-250 (lower panel); **4C** - Results of the native 1% agarose gel electrophoresis of proteins in TBE buffer; the gels were stained with EtBr (upper panel) and Coomassie blue R-250 (lower panel).

The RNA and DNA contents of the intact VLPs formed by the HBc proteins were estimated using a Qubit fluorometer. This analysis revealed a gradual decrease in the RNA content with increases in the truncation from the C-end up to aa 156 of the HBc protein (Figure 4). It is interesting to note that HBc1-152, which has one Arg block, similar to HBc1-156, contained half as much RNA as HBc1-156, which harbours an additional GRSP sequence. All VLP variants contained some amount of DNA, with the highest level in VLPs formed by the non-truncated HBc protein. However, this method of nucleic acid detection did not distinguish encapsidated RNA from contaminant RNA weakly bound to the VLP surface. To distinguish these types of RNA, we used two alternate methods: double radial Ouchterlony immune diffusion using polyclonal rabbit anti-HBc antibodies (Figure 4B) and native agarose gel electrophoresis (Figure 4C). Immunodiffusion showed sharp precipitation bands in all cases, but the presence of nucleic acid in VLPs formed by HBc proteins shorter than 156 aa long (Figure 4B) was not observed, thus generally confirming the Qubit data (Figure 4A). Both methods led to the same conclusion that HBc1-162 was the last C-terminally truncated HBc variant that retained the ability to encapsidate substantial amounts of RNA, although less efficiently than the longer HBc variants. In general terms, for the VLPs formed by HBc variants longer than HBc1-156, the RNA encapsidation ability could be characterised as comparable to that of the full-length HBc1-183.

Roughly, the antigenic identity of all HBc VLPs was confirmed by the fact that the normalised (to 1 mg/mL) HBc VLP samples, including full-length HBc1-183, had similar 1:32-1:64 titres in the Ouchterlony immunodiffusion test, which could be regarded as the most specific test for VLP standardisation and quality assessment because it can discriminate unassembled HBc proteins. It is surprising that the precipitation lines formed by the C-terminally truncated HBc VLPs flowed together with the precipitation line produced by the full-length HBc1-183 VLPs without any “spurs” (Figure 4B). These results demonstrated the full “Ouchterlony identity” of the HBc VLPs tested, even though they differed substantially with respect to molecular mass and RNA content.

As well as Ouchterlony test, NAGE (native agarose gel electrophoresis) did not reveal crucial differences in the stability of the VLP preparations. HBc1-183 appeared to be the most stable in NAGE, showing less smear in the gel. In other cases, the smear along the track was more or less visible both in Coomassie- and EtBr-stained gels (Figure 4C), with some of the HBc material remaining at the start position. Smears at some extent were also observed for VLP samples containing background levels of nucleic acid.

**Figure 5:**
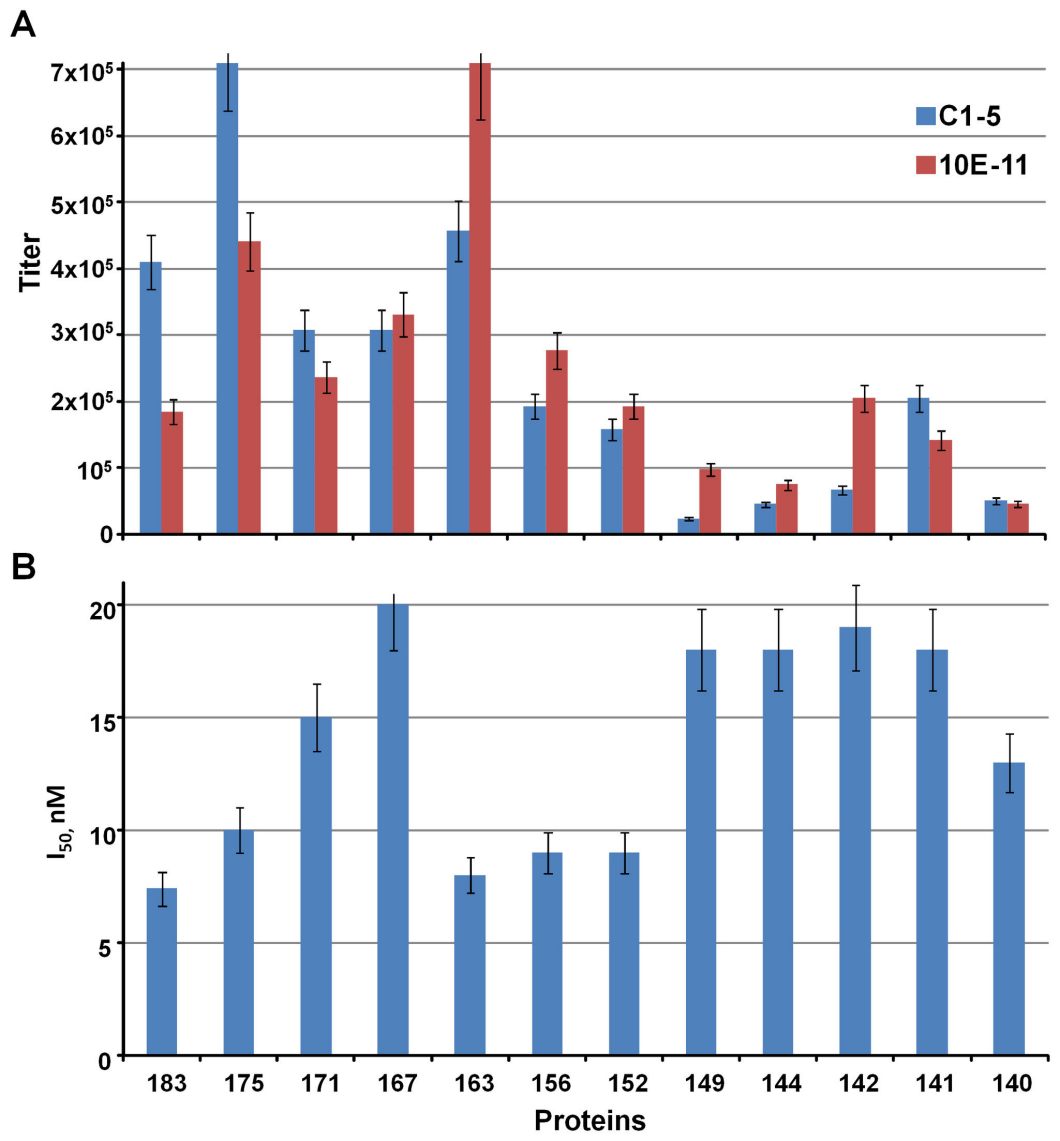
Antigenicity of the VLPs formed by the HBc protein variants. **5A** - direct and **5B** - competitive ELISA data characterizing reactivities of the different HBc proteins to mAbs C1-5 [46] and 10E11 [44]. The results represent the mean values (± SD) of three separate experiments.

In our previous publication, we have chosen the thermal stability as a measure of the VLP stability [47]. Here, we did away with this parameter, since the latter cannot explain processes, which do occur at the constant physiological temperature. As a result, we have chosen a test of the accessibility to monoclonal antibodies of the HBc particles on the solid support as an objective measure of the VLP stability (Figure 5).

The direct ELISA showed very similar results for the VLPs formed by HBc proteins longer than 162 aa, with no significant differences when using mAb C1-5, which recognises an “outer” immunodominant epitope, 78-DPIxxD-83, at the tips of the core spikes [46], or 10E11, which recognises the N-terminal part of HBc [44] (Figure 5A). As for the shorter HBc variants, the ELISA results were less reproducible, with a tendency of lower reactivity with both mAbs. We attribute this result to the lower affinity for the mAbs of the VLPs formed by the short HBc proteins due to the conformational changes and thus lower stability of VLPs on solid support caused most likely by the absence of encapsidated nucleic acids. The inhibitory concentrations (I_50_) in a competitive ELISA (Figure 5B) were in the range of 7-20 nM for different VLPs and did not differ significantly from the I_50_ value for the full-length HBc1-183 (7 nM). The possibility of definite differences in the structure of the C-terminally truncated HBc VLPs cannot be excluded because such differences have been found between bacterially expressed RNA-containing HBc particles and mature DNA-containing HBc particles extracted from virions [23].

**Figure 6:**
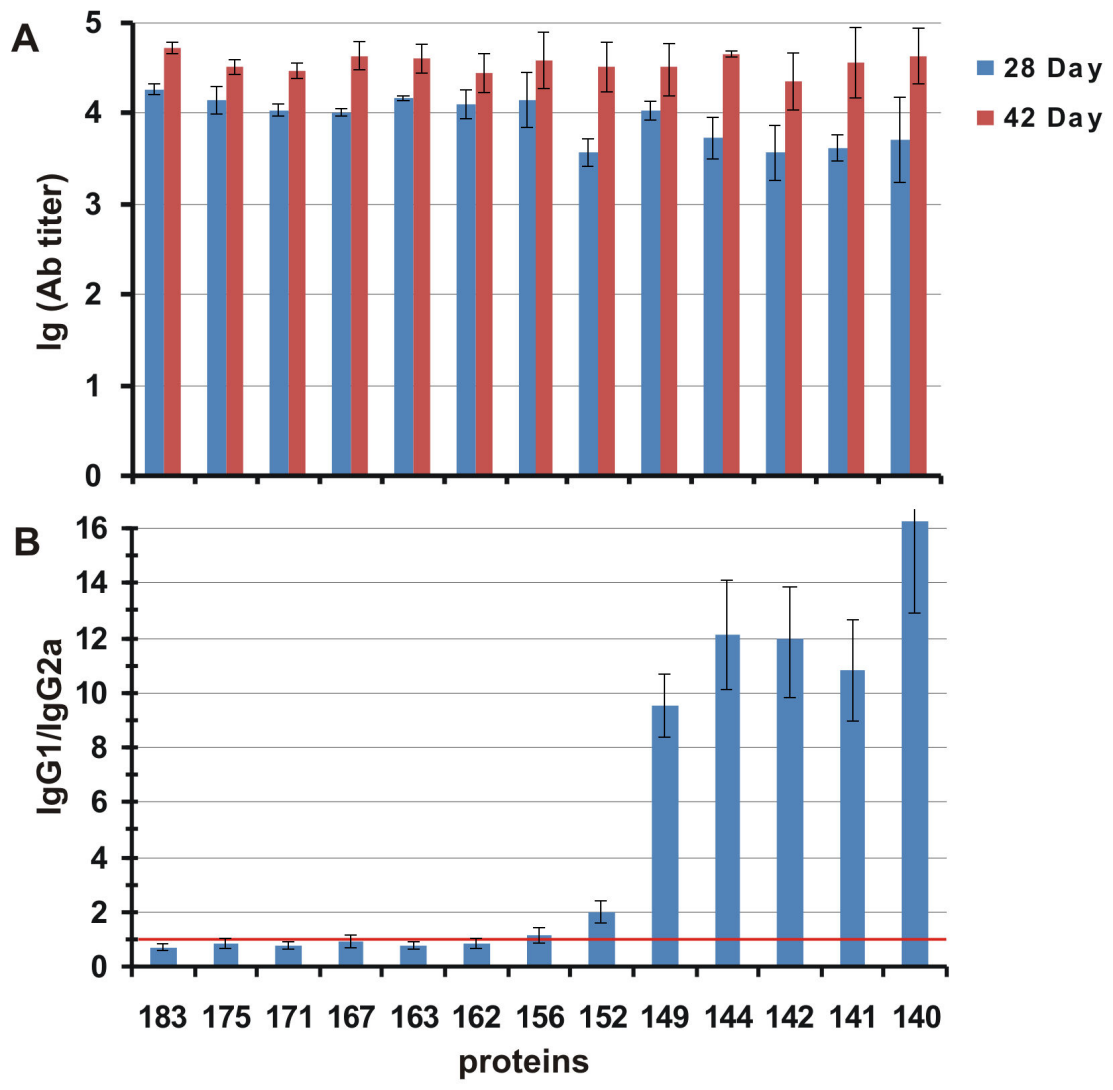
Immunogenicity of the HBc proteins. **6A** - Average antibody titres for the sera from five animals are shown. The recombinant full-length HBc1-183 protein was used to coat the solid phase. The results represent the mean values (± SD) of five individual mouse sera; **6B** - Average IgG isotype profile in mouse sera from five animals after the immunisation with VLPs formed by HBc proteins of different lengths. The red line depicts the equivalent distribution of the IgG2a and IgG1 isotypes in the sera. The results depicted represent the mean values (± SD) of five individual mouse sera.

We were not able to find significant differences in the antibody responses of mice among the VLPs formed by different HBc proteins; in all cases, the antibody titres were in the range of 2-4 x 10^4^ (Figure 6A). On day 28, the responses were several times lower for the VLPs formed by HBc1-183 and truncated variants up to 1-156 aa. The anti-HBc antibody-specific isotype profiles are presented in Figure 6B. The substantial contribution of the IgG2a isotype (the IgG1/IgG2a ratio was in the range of 0.2 to 1) in the sera was typical for VLPs formed by HBc proteins longer than 152 aa and reached up to a 16-fold predominance of IgG1 over IgG2a for shorter HBc VLPs. Therefore, the switching of the IgG1/IgG2a ratio occurred as a result of the elimination of aa residues beyond position 152. The IgG1/IgG2a isotype ratio of the anti-HBc antibodies revealed a strong correlation between Th1 priming and the presence of encapsidated RNA. As it has been found earlier, HBe antigen, a non-particulate RNA-free analogue of HBc antigen [48], as well as recombinant RNA-free HBc1-144 and HBc1-149 variants from bacteria [36] primed predominantly Th2 immunity, in opposite to full-length RNA-containing HBc1-183, which primed predominantly Th1 immunity. The same Th1/Th2 switch has been described by us for full-length and C-terminally truncated HBc variants carrying HBV preS1 [49] and HCV [47] epitopes.

It has been demonstrated previously that HBc is recognized by B cells rather than by non-B cell professional antigen-presenting cells such as dendritic cells or macrophages [50,51]. Although a role of HBc C-terminus in direct binding to cell membrane heparan sulphates has been suggested [39,52], the presence of encapsidated single-stranded RNA bound to the C-terminus of the full-length HBc1-183 from *E. coli* or yeast has been connected categorically with the predominant Th1 immunity [36]. Finally, it was demonstrated at the T and B cell levels in TLR-7 knockout mice that bacterial, yeast, and mammalian single-stranded RNAs encapsidated within full-length HBc1-183 do function as TLR-7 ligands [53]. Here, we show that C-terminally truncated HBc variants, which retain their ability to encapsidate RNA, demonstrate the same Th1 immunity profiles as initial HBc1-183, irrespective of the length of the C-terminus.

**Figure 7:**
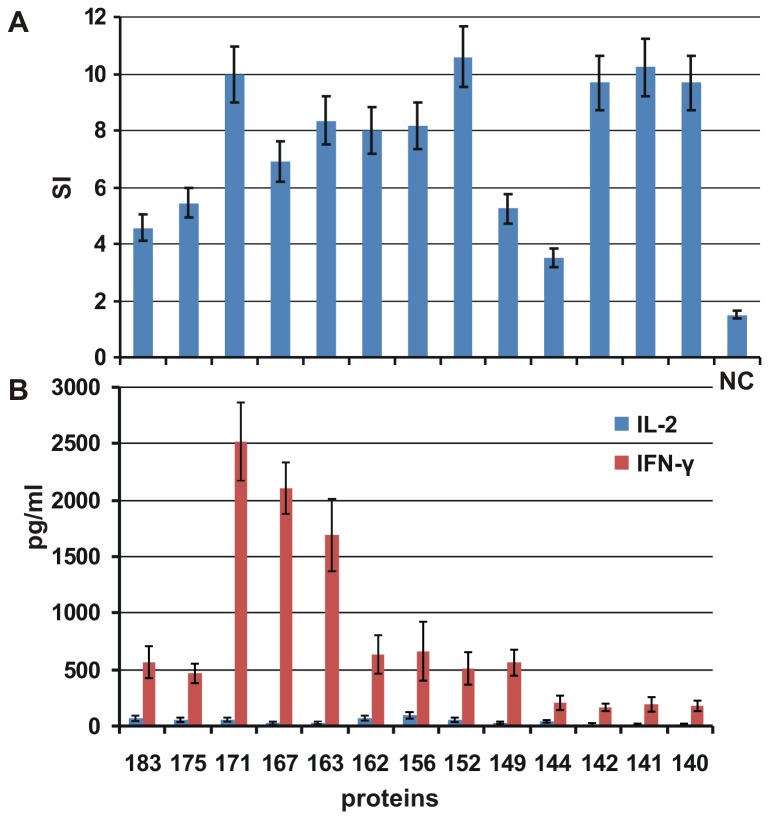
T-cell proliferation after the immunisation of Balb/c mice with VLPs formed by different HBc proteins. **7A** - Stimulation indexes (SI) were calculated for the responses of T cells to stimulation with full-length HBc. NC - control group or naïve (unstimulated) mice. The results depicted represent the mean values (± SD) of two parallel calculations. **7B** - Cytokines produced by T cells after the immunisation of Balb/c mice with VLPs formed by different HBc proteins. Culture supernatants from cells stimulated with full-length HBc were removed and analysed at 24 h and 48 h to assess IL-2 and IFN-γ production, respectively. The results represent the mean values (± SD) of two parallel experiments.

T-cell proliferation and the IL-2 and IFN-γ levels were detected in the lymphocytes from spleens (obtained on day 42) of mice immunised with VLPs formed by full-length and C-terminally truncated HBc protein variants. T cells were stimulated *in vitro* using full-length HBc1-183, and the appropriate stimulation indexes were determined (Figure 7A). Clear proliferation was detected in all cases, without any correlation with the length of the HBc protein forming the VLPs.

The highest level of IFN-γ produced after stimulation with HBc1-183 was detected for mice immunised with VLPs formed by middle-sized variants of HBc: HBc1-171, HBc1-167, and HBc1-163. The production of IL-2 was low in all cases. The decrease in IFN-γ production correlated approximately with the decrease in the relative IgG2a level in the sera from mice immunised with VLPs formed by the shortest forms of HBc (with aa 1-149 or less, Figure 6B). The decrease in IFN-γ synthesis for the HBc variants with C-truncations beyond aa 149 could be speculatively explained by the loss of a potential CTL epitope at 141-151 [54]. However the reasons for the lower levels of both IL-2 and IFN-γ for the full-length protein HBc1-183 and HBc1-171 remain unclear.

The present investigation developed a set of novel HBc vectors that can be used not only for the generation of traditional vaccine candidates and cell targeting tools that expose foreign peptide stretches on the HBc surface but also for the selective packaging of different substrates. The C-terminally truncated HBc variants may differ in their affinity for substrates, inner space, packaging capacity, and ability to dissociate/re-associate, which makes them attractive tools for the further development of HBc-based gene and drug delivery applications. Although the HBc protein exhibits high-level synthesis and efficient self-assembly in virtually all known homologous and heterologous expression systems [55], *E. coli* remains the most attractive system when efficient expression and high yields of recombinant HBc proteins are necessary.
